# Exercise Management Using a Mobile App in Patients With Parkinsonism: Prospective, Open-Label, Single-Arm Pilot Study

**DOI:** 10.2196/27662

**Published:** 2021-08-31

**Authors:** Aram Kim, Seo Jung Yun, Kwan-Sik Sung, Yeonju Kim, Ju Young Jo, Hanseul Cho, Kyudong Park, Byung-Mo Oh, Han Gil Seo

**Affiliations:** 1 Department of Rehabilitation Medicine Seoul National University College of Medicine Seoul National University Hospital Seoul Republic of Korea; 2 College of Medicine Kyung Hee University Seoul Republic of Korea; 3 School of Information Convergence Kwangwoon University Seoul Republic of Korea; 4 Institute on Aging Seoul National University Seoul Republic of Korea; 5 National Traffic Injury Rehabilitation Hospital Yangpyeong Republic of Korea

**Keywords:** Parkinsonian disorders, exercise, mobile apps, mhealth, Parkinson

## Abstract

**Background:**

Patients with parkinsonism have higher inactivity levels than the general population, and this results in increased comorbidities. Although exercise has benefits for motor function and quality of life (QOL) in patients with parkinsonism, these patients face many barriers to exercise participation, such as lack of motivation, fatigue, depression, and time constraints. Recently, the use of mobile apps has been highlighted as a remote exercise management strategy for patients with chronic diseases.

**Objective:**

This study aimed to evaluate the effects of home-based exercise management with a customized mobile app on the exercise amount, physical activity, and QOL of patients with parkinsonism.

**Methods:**

This was a prospective, open-label, single-arm pilot study. The therapist installed the app in the smartphones of the participants and educated them on how to use the app. The therapist developed an individualized multimodal exercise program that consisted of stretching, strengthening, aerobic, balance and coordination, and oral-motor and vocal exercises. Participants were encouraged to engage in an 8-week home-based exercise program delivered through a customized app. The alarm notifications of the app provided reminders to exercise regularly at home. The primary outcome was the exercise amount. The secondary outcomes were assessed using the International Physical Activity Questionnaire (IPAQ), Parkinson’s Disease Questionnaire-39 (PDQ-39), and Geriatric Depression Scale (GDS). The usability of the customized app was assessed using a self-report questionnaire.

**Results:**

A total of 21 participants with parkinsonism completed the intervention and assessment between September and December 2020 (mean age: 72 years; women: 17/21, 81%; men: 4/21, 19%). The participants reported a significant increase in the total amount of exercise (baseline: mean 343.33, SD 206.70 min/week; 8-week follow-up: mean 693.10, SD 373.45 min/week; *P*<.001) and in the amount of each exercise component, including stretching, strengthening, balance and coordination, and oral-motor and vocal exercise after 8 weeks. Analysis of the secondary outcomes revealed significant improvements in the IPAQ (*P*=.006), PDQ-39 (*P*=.02), and GDS (*P*=.04) scores. The usability of the program with the mobile app was verified based on the positive responses such as “intention to use” and “role expectation for rehabilitation.”

**Conclusions:**

Exercise management with a customized mobile app may be beneficial for improving exercise adherence, physical activity levels, depression management, and QOL in patients with parkinsonism. This remotely supervised technology-based, reinforcing, and multimodal exercise management strategy is recommended for use in patients with parkinsonism. In addition, this program proved useful as an alternative exercise management strategy during the COVID-19 pandemic when patients with Parkinson disease were less physically active than before and showed aggravation of symptoms. However, additional clinical trials are needed to evaluate the efficacy of this exercise program in a large population and to confirm its disease-modifying effects.

## Introduction

Parkinson disease (PD) is a progressive neurodegenerative disease characterized by motor symptoms including tremors, rigidity, and bradykinesia, and nonmotor symptoms such as depression and cognitive impairment [1]. The physical activity level of patients with PD is approximately one-third of the general population because of their physical, cognitive, and emotional impairments. A previous study investigated the determinants of physical inactivity in detail and reported that disease severity, walking impairments, and disability in daily life are factors associated with physical inactivity [2]. Physical inactivity results in increased comorbidities such as cardiovascular events, diabetes mellitus, cancer, and osteoporosis [3]. This is also probably true in patients with atypical parkinsonism, such as multiple system atrophy (MSA), progressive supranuclear palsy (PSP), and corticobasal degeneration (CBD), which show clinical symptoms similar to those of PD and whose differential diagnosis can be challenging in the early disease stages [4].

Numerous studies have shown the positive effects of physical activity in terms of preventing depression and cognitive decline, as well as for improving strength, balance, and flexibility in patients with PD [5-7]. With respect to the type of exercise, considerable evidence suggests the benefits of daily walking, strength training, and Tai Chi. In addition, swimming, cycling, and dancing are reported to have physical benefits. Although there is insufficient evidence to prove the superiority of one type of exercise over others, owing to heterogeneity in the details of the exercise and outcome variables in previous studies, regular exercise is known to improve motor function and quality of life (QOL) in patients with PD regardless of the exercise type [8]. Tomlinson et al [9] reviewed the short-term benefits of a physical intervention program on gait, balance, and disability outcomes, and Mak et al [10] reviewed the long-term benefits of exercise on motor symptoms and physical function parameters such as muscle strength, aerobic capacity, gait impairment, balance, and fall risk. In particular, long-term improvements caused by exercise may indicate a disease-modifying effect, which has been proven with respect to progressive resistance, aerobic, and balance training in human and animal model studies [11,12]. Although only a few studies have been published on the effect of exercise on atypical parkinsonism, the benefits of several types of exercise have been reported in patients with MSA, PSP, and CBD [13-15].

Exercise is recommended at all disease stages, regardless of active or sedentary states [7,16]. Even active patients face several barriers (eg, lack of motivation, fatigue, depression, and time constraints) to exercise participation, especially to long-term exercise maintenance [17]. An effective exercise management strategy for patients with PD needs to be focus on providing motivational and enjoyable experiences to facilitate regular exercise. With respect to the type of exercise management strategy, previous studies comparing center-based and home-based exercise programs in older adults have found that center-based exercises are superior in the short term and home-based exercises are superior in the long term with respect to adherence [18]. A previous study reported that minimally supervised home-based programs using collaborative goal setting between therapists and patients, exercise diary records, and intermittent follow-ups can improve adherence and achieve sustained improvements in patients with PD [19]. Recently, the use of specially developed mobile apps has been highlighted as a remote exercise management strategy for patients with chronic diseases. Owing to the increasing life expectancy in patients with chronic diseases, maintaining QOL is important and management using mobile apps has been proven to improve outcomes in this population [20]. In addition, contactless methods of health management are emerging owing to unexpected situations such as the COVID-19 pandemic, and elderly people with chronic disease are the most vulnerable. The use of mobile apps can be an alternative exercise management strategy in patients with PD during the COVID-19 pandemic.

This study aimed to evaluate the effects of a minimally supervised home-based exercise program delivered through a customized mobile app on the exercise amount, physical activity, emotional well-being, and QOL in patients with parkinsonism. We also aimed to assess the usability of the mobile app to provide a basis for further studies on the broad application of mobile apps in future.

## Methods

### Participants

Participants were recruited from the outpatient rehabilitation clinic of a tertiary hospital. The inclusion criteria were as follows: (1) diagnosed with PD or atypical parkinsonism conditions such as MSA, PSP, and CBD; (2) aged above 46 years; and (3) regular participation in a PD exercise program at least once a week. The exclusion criteria were as follows: (1) severe cognitive or physical impairment (Hoehn and Yahr stage 5) interfering with participation in the exercise program during this study and (2) no requirement for exercise management because the amount of recommended exercise was already being performed (>3 h/d in patients at Hoehn and Yahr stages 1-2 and >2 h/d in patients at Hoehn and Yahr stages 2.5-4). The adequate sample size was defined as 24 considering a 20% dropout rate for a pilot study. This study was approved by the institutional review board of our hospital on August 28, 2020 (approval no. 2007-157-1144), and written informed consent was obtained from all participants. The participants were on common treatment regimens for parkinsonism and maintained their previous exercise programs during the study.

### Intervention

**Figure 1:**
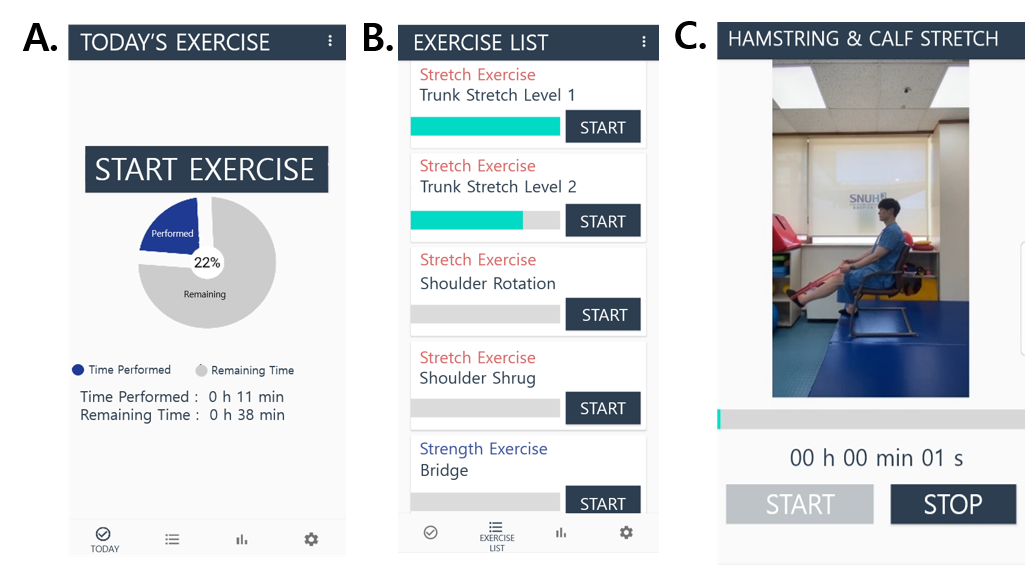
Example of an individualized exercise program using a mobile app: (A) goal and degree of completion for the day, (B) list of exercise components, and (C) video-guided exercise technique. (For the convenience of readers, the text in the figure was translated into English [originally in Korean]).

The components of the exercise program were stretching, strengthening, aerobic, balance and coordination, and oral-motor and vocal exercises. The customized app showed the goal (total exercise amount) for the day, a detailed list and the duration for each component, and a video-guided exercise technique recorded by experienced physical and occupational therapists (Figure 1). The alarm notifications for the exercise were initially set by a therapist and later changed by the participants depending on their circumstances. The alarm notifications of the app provided reminders to maintain regular exercise and messages to motivate the patient. In addition, when the patient clicked the button indicating the completion of one exercise component, the app showed the next exercise component to encourage the patient to continue exercising. The app was designed to provide elements of accomplishment and pleasure to promote patient adherence.

**Figure 2:**
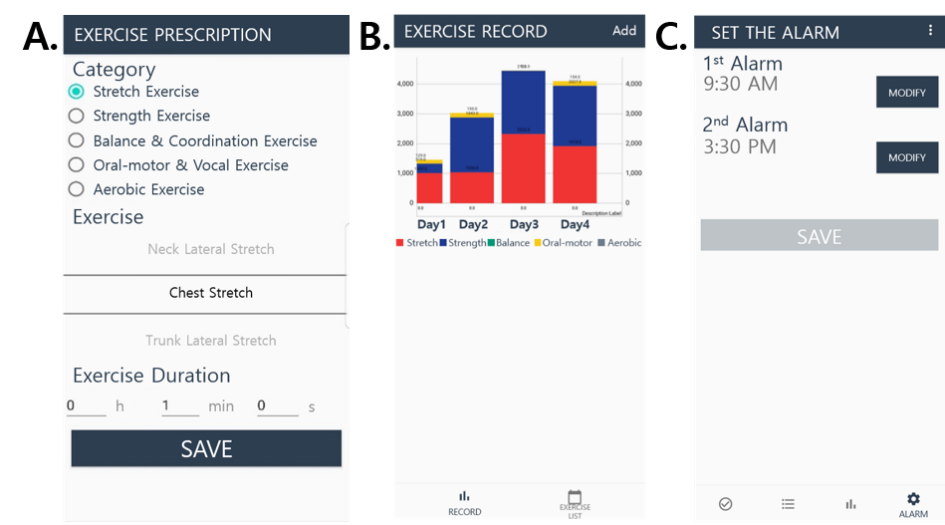
Example of management by the professional therapist: (A) set of exercise components (type, duration), (B) record of the amount of exercise for monitoring, and (C) set off alarms. (For the convenience of readers, the text in the figure was translated into English [originally in Korean]).

Before starting the exercise program, the therapist installed the app on the personal smartphones of the participants and educated them on how to use the app. The therapist developed an individualized exercise program that consisted of multimodal exercises based on European physiotherapy guidelines for PD [21]. The exercise amount was monitored, and the exercise program and notification time were regularly adjusted by the therapist according to the preference and compliance of each participant (Figure 2).

### Outcome Measures

Baseline characteristics including the age, sex, diagnosis, disease duration, current medications, Hoehn and Yahr stage, Berg Balance Scale (BBS) score, and timed up-and-go (TUG) test time were assessed. The daily dose of each antiparkinsonian drug was converted into the levodopa equivalent dose (LED), which is a useful indicator of the drug intensity of different medications [22]. The Hoehn and Yahr scale describes the severity of PD from stage 0 through stage 5 [23]. The BBS is a widely used tool for assessing balance performance, in which a higher score indicates better balance ability and a score of 45 points was suggested as the cutoff value for independent ambulation [24]. The TUG test assesses functional mobility, in which a time of <10 seconds means normal mobility and 11–20 seconds is the normal range in elderly patients and patients with disabilities [25].

The outcome measures were assessed at baseline and after using the app for 8 weeks. The assessments included self-completed questionnaires and interviews providing information on exercise amount, physical activity, depression, and QOL.

As the primary outcome, the exercise amount was estimated in terms of the frequency (numbers per week) and duration (minutes per day), and the total amount of exercise was calculated by multiplying the frequency and duration for all exercises and each component. In addition, subjective intensity (Borg scale 6-20) was assessed for the total exercise amount.

To measure physical activity, we used the International Physical Activity Questionnaire (IPAQ), which has been used in the World Health Organization’s World Health Survey. The IPAQ questionnaire comprises 7 questions about the frequency and duration of vigorous activity, moderate activity, walking, and sitting. The total physical activity was calculated by multiplying the time (minutes per week) by the intensity (metabolic equivalent of task [MET] unit), and classified as insufficient activity (<600 MET), sufficient activity (600–1499 MET, or vigorous activity for ≥20 minutes on ≥3 days, or moderate activity and walking for ≥30 minutes on ≥2 days), and high activity (1500–2999 MET with ≥3 days of vigorous activity or >2999 MET with ≤2 days of vigorous activity) [26]. The validity of the Korean version of the IPAQ instrument has been proven in the Korean population [27,28].

For evaluating depression, the participants were asked to complete the Geriatric Depression Scale (GDS) Short Form. The GDS is a widely used screening tool for depression in old age. The GDS Short Form contains 15 items. A score of 6 to 7 points was suggested as the cutoff value in Western countries, whereas a score of 10 points was recommended as the cutoff point in a Korean validation study [29].

The QOL of patients with PD was assessed using the Parkinson’s Disease Questionnaire-39 (PDQ-39), which is a tool used worldwide and available in many different languages. This tool contains 39 items categorized into 8 dimensions: mobility, activities of daily living, emotional well-being, stigma, social support, cognition, communication, and bodily pain. The total PDQ-39 score is expressed as a percentage out of a total of 100, and a lower score indicates better QOL. The Korean PDQ-39 has been validated in Korean patients with PD [30].

Furthermore, the usability of the customized app was evaluated using a self-report questionnaire including 8 items (symptom improvement, interest, adequate difficulty, physical comfort, stability, satisfaction, intention to use, and role expectations for rehabilitation), with a 7-point Likert-type scale.

### Statistical Analysis

Data were analyzed using SPSS software for Windows (version 25.0; IBM Corp). The normality of the data was tested using the Shapiro–Wilk test. To detect a change in the amount of exercise, IPAQ, GDS, and PDQ-39 between baseline (T0) and after 8 weeks (T1), paired *t* tests, and Wilcoxon signed rank tests were used. The level of significance was set at *P*<.05.

## Results

### Patient Characteristics

Among 28 patients with parkinsonism who were initially screened between September and December 2020, 24 met the inclusion criteria. Two participants withdrew because of fractures associated with a fall event during daily activities, and one participant requested to discontinue the program because of difficulty in using the app. Therefore, a total of 21 participants completed the intervention and assessment.

The characteristics of the participants at baseline are presented in Table 1. The disease severity was moderate to severe, corresponding to Hoehn and Yahr stages 2- 4. In this study, the BBS score and TUG test time ranged from 21 to 56 points and from 13.87 to 7.82 seconds, respectively. All participants were on common dopaminergic treatment (LED: mean 663.94, SD 357.79).

**Table 1:** Baseline characteristics of participants (N=21).

Participant characteristics			Value
Age (years), mean (SD); range			72.38 (5.77); 62-82
**Sex, n (%)**			
	Female	17 (81)	
	Male	4 (19)	
**Diagnosis, n (%)**			
	Parkinson disease	13 (61.9)	
	Atypical parkinsonism	8 (38.1)	
Disease duration (years), mean (SD); range			8.14 (4.95); 2-18
**Hoehn and Yahr scale (stage), n (%)**			
	2	1 (4.8)	
	2.5	8 (38.1)	
	3	8 (38.1)	
	4	4 (19)	
Berg Balance Scale score (points), mean (SD); range			48.38 (9.84); 21-56
Timed up-and-go test time (s), mean (SD); range			13.87 (7.82); 7-42
Levodopa equivalent dose, mean (SD); range^a^			663.94 (357.79); 300-1613

^a^n=18, excluding 3 participants without information on current medications from other medical centers.

### Exercise Amount

The participants showed a significant increase of almost 2 times in the total exercise amount per week (T0: mean 343.33, SD 206.70 min/week; T1: mean 693.10, SD 373.45 min/week; *P*<.001) after the study intervention. The amount significantly increased for all exercise components (stretching, strengthening, balance and coordination, oral-motor, and vocal exercises), except for aerobic exercise. A greater increase in the amount of exercise per week was observed in the strengthening component (T1-T0: mean 107.62, SD 83.38 min/week; *P<.*001), followed by the stretching (T1–T0: mean 83.81, SD 188.98 min/week; *P*=.04), balance and coordination (T1-T0: mean 56.90, SD 63.81 min/week; *P*<.001), and oral-motor and vocal (T1-T0: mean 24.38, SD 42.23 min/week; *P*=.01) components. Furthermore, the subjective intensity of exercise (Borg scale 6-20) also increased after 8 weeks of using the app (Table 2).

**Table 2:** Results for exercise amount, the primary outcome.

Parameter		Baseline (T0)	8 weeks (T1)	Within-individual change (T1-T0)	*P* value
**Frequency (number/week), mean (SD)**					
	Stretching	5.14 (1.93)	5.57 (1.47)	0.43 (2.31)	.53
	Strengthening	1.43 (2.54)	5.14 (1.96)	3.71 (2.9)	*<.001^a^*
	Aerobic	5.05 (1.71)	5.19 (1.66)	0.14 (1.98)	.75
	Balance and coordination	1 (1.73)	3.71 (2.59)	2.71 (3.16)	*.002*
	Oral-motor and vocal	2.9 (2.83)	3.9 (2.81)	1 (2.14)	*.045*
	Total	5.33 (1.65)^a^	5.86 (1.32)	0.52 (1.66)	.16
**Duration (min/day), mean (SD)**					
	Stretching	17.95 (13.29)	31.52 (29.56)	13.57 (31.71)	.07
	Strengthening	3.81 (7.57)	22.62 (14.88)	18.81 (15.64)	*<.001*
	Aerobic	42.14 (21.94)	44.29 (23.36)	2.14 (26.3)	.71
	Balance and coordination	5.48 (8.65)	18.10 (16.84)	12.62 (17.51)	*.004*
	Oral-motor and vocal	7.62 (8.31)	12.00 (13.75)	4.38 (12.15)	.07
	Total	61.43 (28.16)	115.48 (54.63)	54.05 (52.86)	*<.001*
**Frequency × duration (min/week) **					
	Stretching	96.86 (81.25)	180.67 (178.40)	83.81 (188.98)	*.04*
	Strengthening	15.95 (28.18)	123.57 (89.99)	107.62 (83.38)	*<.001*
	Aerobic	211.67 (124.03)	245.24 (169.90)	33.57 (155.07)	.33
	Balance and coordination	13.81 (22.47)	70.71 (67.80)	56.90 (63.81)	*.001*
	Oral-motor and vocal	34.76 (39.73)	59.12 (59.75)	24.38 (42.23)	*.01*
	Total	343.33 (206.70)	693.10 (373.45)	349.76 (344.54)	*<.001*
Intensity (Borg 6–20)		11.86 (1.74)	13.14 (1.42)	1.29 (2.08)	*.02*

^a^Italicized values indicate statistical significance.

### Physical Activity, Emotional Well-Being, and QOL

**Figure 3:**
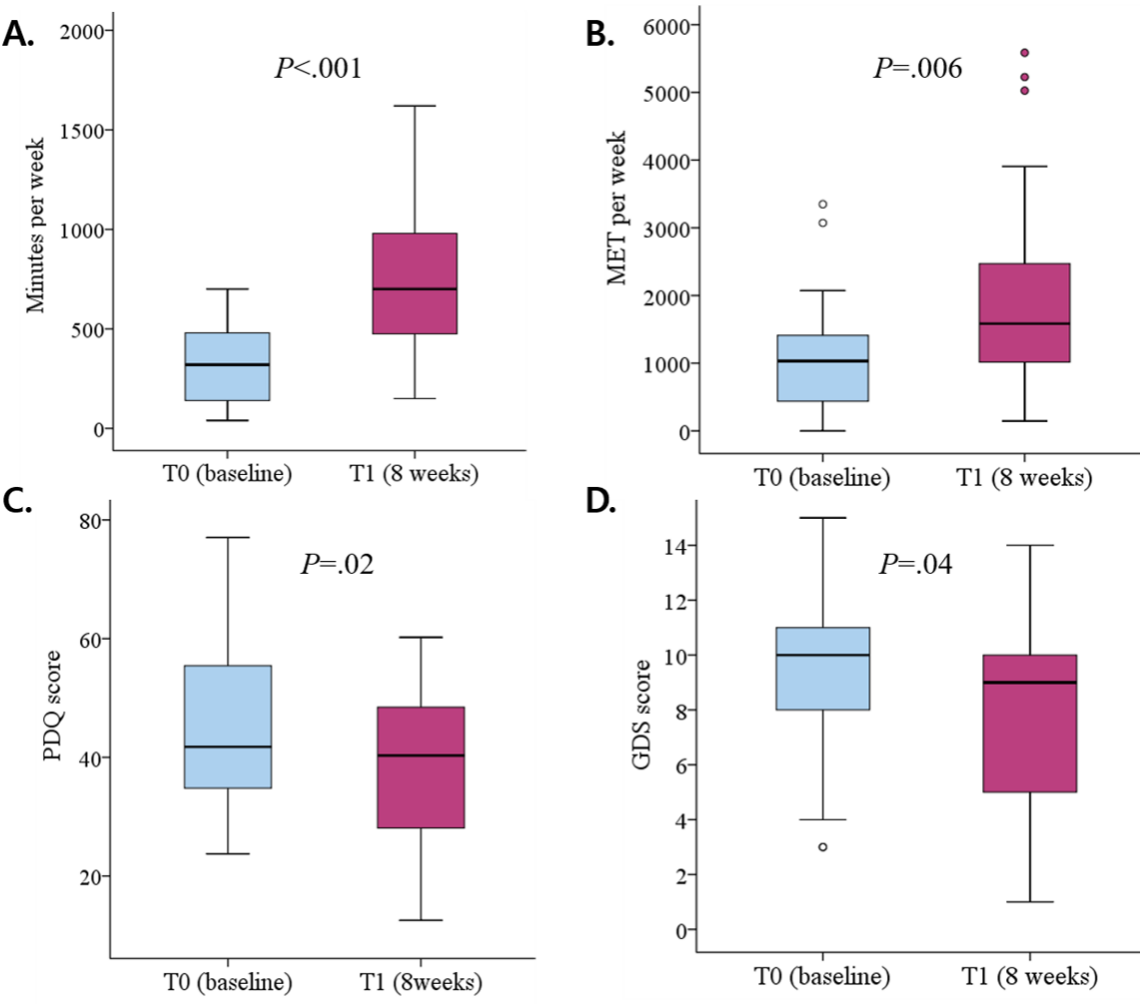
Overview of the changes after participating in the home-based exercise program using a mobile app: (A) total exercise amount, (B) MET score measured using the IPAQ, (C) PDQ-39, and (D) GDS score. GDS: Geriatric Depression Scale; IPAQ: International Physical Activity Questionnaire; MET: metabolic equivalent of task; PDQ: Parkinson Disease Questionnaire.

Most participants showed IPAQ scores corresponding to a sufficient activity level at baseline, which indicated that they were active rather than sedentary. We observed a significant increase of almost 2 times in the IPAQ score (T0: mean 1104.17, SD 911.63 MET/week; T1: mean 2027.17, SD 1636.38 MET/week; *P*=.006), classified as high activity, after the intervention. A statistical trend toward a decrease in the time associated with the sedentary state was also observed. In addition, our findings showed that the GDS and PDQ-39 scores were lower at T1 than at T0, indicating significant improvements in depression and QOL (Table 3). Figure 3 shows an overview of the changes in the total amount of exercise, IPAQ score, PDQ-39 score, and GDS score.

**Table 3:** Results for the secondary outcomes determined using the International Physical Activity Questionnaire, Parkinson Disease Questionnaire-39, and Geriatric Depression Scale.

Parameter		Baseline (T0)	8 weeks (T1)	Within-individual change (T1-T0)	*P* value
**IPAQ score^a^ (MET/week), mean (SD)**					
	Vigorous activity	91.43 (418.98)	480.00 (1204.79)	388.57 (1036.39)	.10
	Moderate activity	435.24 (578.89)	636.52 (616.20)	201.29 (596.41)	.14
	Walking	577.5 (386.68)	910.64 (605.66)	333.14 (488.82)	*.005*
	Sedentary	370.48 (196.02)	290 (104.5)	–80.48 (190.17)	.07
	Total	*1104.17 (911.63)* ^b^	*2027.17 (1636.38)*	*923.00 (1406.38)*	*.006*
**PDQ-39^c^ score (points), mean (SD)**					
	Mobility	54.76 (22.76)	49.52 (21.28)	–5.24 (21.88)	.29
	Activities of daily living	44.25 (28)	38.69 (27.8)	–5.56 (19.82)	.21
	Emotional well-being	47.02 (19.51)	46.63 (22.85)	–0.4 (20.07)	.93
	Stigma	37.5 (16.06)	30.65 (14.38)	–6.85 (21.28)	.16
	Social support	35.71 (20.44)	37.3 (20.69)	1.59 (21.18)	.74
	Cognition	41.67 (19.4)	36.9 (17.67)	–4.76 (10.81)	.06
	Communication	37.7 (29.42)	31.75 (27.6)	–5.95 (16.06)	.11
	Bodily pain	53.97 (18.93)	36.9 (26.43)	–17.06 (21.49)	*<.001*
	Total	44.07 (14.57)	38.54 (14.23)	–5.53 (10.26)	*.02*
GDS^d^ score (points)		9.48 (3.42)	7.86 (3.7)	–1.62 (3.25)	.04

^a^IPAQ: International Physical Activity Questionnaire.

^b^Italicized values indicate statistical significance.

^c^PDQ-39: Parkinson Disease Questionnaire-39.

^d^GDS: Geriatric Depression Scale.

### Usability

With respect to usability according to a 7-point Likert-type scale, the highest score was observed for “intention to use” (mean 6.14, SD 0.77), followed by “role expectation for rehabilitation” (mean 6.10, SD 0.83), “stability” (mean 5.48, SD 1.22), “satisfaction” (mean 5.28, SD 1.03), “interest” (mean 5.24, SD 1.06), “adequate difficulty” (mean 5.24, SD 1.02), “physical comfort” (mean 4.48, SD 1.26), and “symptom improvement” (mean 4.23, SD 0.97).

## Discussion

### Principal Results

The results of this study indicated a significant increase in the amount of exercise and each component (except for aerobic exercise) after 8 weeks of exercise management using a customized mobile app in patients with parkinsonism. The motivational app had an additional benefit even for participants who had been active before participating in the exercise program. Moreover, we observed significant improvements in physical activity, depression, and QOL at the 8-week follow-up assessment.

The primary outcome of this study was the exercise amount, which represented adherence by patients with parkinsonism to the home-based exercise program with a customized app. Earlier studies have evaluated the effectiveness and usability of tablet-based apps in patients with PD. van der Kolk et al [31] investigated a home-based and remotely supervised aerobic exercise program delivered through a customized tablet-based app (for exercise instructions and monitoring), and they found improvements in the disease severity based on the Unified Parkinson’s Disease Rating Scale (UPDRS) motor score [31]. Siegert et al [32] reported their study protocol for a home-based exercise program using an app installed on a tablet to reinforce health behaviors, established by completing a 3-week center-based exercise program. Furthermore, a few recent studies have investigated the efficacy, feasibility, and safety of using mobile app–based exercise programs. Landers and Ellis [33] reported a single-cohort pilot study on the use of a commercially available app with a video-guided exercise program for patients with PD. The exercise program was set through an automatic algorithm depending on the function level of the patient and assessed using demographic questions and remote performance-based tests without professional support. The feasibility, safety, and efficacy were proven; however, a high dropout rate was reported. This suggests that “supervised” management by professionals may be necessary for developing an individualized exercise program and for maintaining the patients’ adherence to exercise. Ellis et al [34] reported a 12-month randomized controlled pilot study evaluating the effectiveness of mobile health–supported exercise programs compared with only conventional exercise programs in patients with PD. The participants were provided an iPad with an app containing a video-guided exercise prescription, and the exercise prescription was adjusted depending on the remotely monitored compliance. This study found no significant difference between groups in terms of improvements in physical activity, although the mobile health–supported exercise program had more benefits for less active participants (completing <7500 steps/day). Our study can contribute to this growing area of research by exploring the feasibility and efficacy of remotely supervised technology-based reinforcing exercise programs in relatively active patients with parkinsonism. Our findings, although preliminary, suggest that a home-based exercise program using a customized mobile app may be an appropriate strategy for improving compliance in patients with parkinsonism.

In this study, a statistical trend toward an increase in the amount of the aerobic exercise component was observed; however, it was not statistically significant compared to the changes in the other exercise components. A possible explanation may be that aerobic exercise generally does not require a specific technique or instruction. Moreover, as shown in Table 2, aerobic exercise was performed for a relatively longer duration than others, and the participants were already performing aerobic exercise for over 40 min/d at baseline assessment. A long-term and larger study probably would provide significant results on the aerobic exercise component. Conversely, we found the greatest change in the amount of the strengthening component, followed by the stretching and balance components in this study. These results suggested that the customized app was useful for facilitating exercise components that require a specific technique. Many previous studies have recommended multimodal physical activity (a combination of exercise modalities) rather than a single exercise component [8,10]. In particular, strengthening involving the extensor muscles of the hip and trunk, stretching exercises for the flexor and axial muscles, and balance training for individuals with a high fall risk have been recommended [7]. The video-guided instructions set in the app may provide the proper techniques for all the exercise components, resulting in multimodal physical activity.

Another important finding was the improvement in physical activity observed after 2 months of the exercise program. Moreover, a tendency toward decreased sedentary times was also observed. Previous studies have reported several major barriers that lead to sedentary behavior in patients with PD, including lack of motivation, fatigue, depression, low outcome expectation from exercise, lack of time, fear of falling, and low self-efficacy [17,35]. These findings may suggest the need for a management strategy that aims to improve motivation and adherence. Herein, we propose an exercise program with several benefits, as it is cost-effective, provides alarm notifications, is supervised by professionals, offers collaborative goal setting, and allows intermittent monitoring. This strategy focused on aspects such as motivation, accessibility, and compliance, and alarm notifications emerged as the most significant factor in improving adherence. Furthermore, improvements in depression and QOL were observed in this study. The increased physical activity level might have caused a decrease in depressive symptoms and an improvement in QOL of the study participants [5].

Recently, the COVID-19 pandemic has become a major barrier for patients with chronic diseases to obtain medical support. In a recent study, the impact of the COVID-19 pandemic on patients with PD was surveyed [36]. The study found that patients with PD experienced higher COVID-related psychological distress, were less physically active than before, and showed aggravation of symptoms. Although approximately half of the patients were less active than before, no relationship between physical inactivity and psychological distress was established. This type of remotely supervised home-based exercise program using a mobile app may be recommended as an alternative exercise management strategy for patients with parkinsonism during the COVID-19 pandemic.

### Limitations

The major limitation of this study was the instability of the customized app. Some errors occurred during login, video streaming, and calculation of the exercise time, which may have interfered with the participation and maintenance of exercise by the patients. Therefore, the cumulative exercise time recorded in the app was used only for monitoring, and the amount of exercise was assessed using self-completed questionnaires. Further work is required to develop an improved app with stable server management and additional attractive features, such as voice instructions, a bigger screen for the elderly, and sharing of exercise data with family and other app users. Another limitation of the study was that we only evaluated the short-term effects of a home-based exercise program with the motivational app. Recent studies reported that long-term improvements caused by exercise may indicate a disease-modifying effect in humans [10,12]. For example, progressive resistance training in patients with PD has been proven to improve motor signs in off-medication UPDRS motor scores. Other studies suggested that an increase in blood oxygen level–dependent signals in basal ganglia circuits and corticomotor excitability results in experience-dependent neuroplasticity [37]. Further studies are warranted to investigate the long-term effect of an exercise program using a customized app in patients with parkinsonism to prove the disease-modifying effects. Lastly, our study included a relatively small sample size, with mixed disease entities in the participants; moreover, there was no control group subjected to a conventional exercise program. In particular, a randomized controlled trial is essential to clarify the effectiveness of the app. Our ongoing follow-up study needs to include a larger sample, more specific disease entity, and control group based on this pilot study.

### Conclusions

A home-based exercise program with a customized mobile app has beneficial impacts on adherence to exercise as well as physical activity, depression, and QOL in patients with parkinsonism. We recommend this program as an additional management strategy (characterized as a remotely supervised technology-based, reinforcing, multimodal strategy) for patients with parkinsonism. Moreover, this program can be an alternative exercise management strategy for patients with parkinsonism who are less physically active and are experiencing difficulties in accessing medical services during the COVID-19 pandemic. Additional clinical trials are needed to evaluate the efficacy of this program in a large population and confirm the disease-modifying effects of this exercise program.

## Abbreviations

BBS: Berg Balance Scale
CBD: corticobasal degeneration
GDS: Geriatric Depression Scale
IPAQ: International Physical Activity Questionnaire
LED: levodopa equivalent dose
MSA: multiple system atrophy
MET: metabolic equivalent of task
PD: Parkinson disease
PDQ-39: Parkinson Disease Questionnaire-39
PSP: progressive supranuclear palsy
QOL: quality of life
TUG: timed up-and-go
UPDRS: Unified Parkinson’s Disease Rating Scale
